# Alberta wildfire 2016: Apt contribution from anomalous planetary wave dynamics

**DOI:** 10.1038/s41598-018-30812-z

**Published:** 2018-08-17

**Authors:** Vladimir Petoukhov, Stefan Petri, Kai Kornhuber, Kirsten Thonicke, Dim Coumou, Hans Joachim Schellnhuber

**Affiliations:** 1 Potsdam Institute for Climate Impact Research (PIK), Member of the Leibniz Association, P.O. Box 60 12 03, D-14412 Potsdam, Germany; 2 Vrije Universiteit, Amsterdam, Netherlands; 3 Stockholm Resilience Centre, Stockholm University, 10691 Stockholm, Sweden

## Abstract

In May-June 2016 the Canadian Province of Alberta suffered one of the most devastating wildfires in its history. Here we show that in mid-April to early May 2016 the large-scale circulation in the mid- and high troposphere of the middle and sub-polar latitudes of the northern hemisphere featured a persistent high-amplitude planetary wave structure dominated by the non-dimensional zonal wave number 4. The strongest anticyclonic wing of this structure was located over western Canada. In combination with a very strong El Niño event in winter 2015/2016 this favored highly anomalous, tinder-dry and high-temperature conditions at the surface in that area, entailing an increased fire hazard there. This critically contributed to the ignition of the Alberta Wildfire in May 2016, appearing to be the costliest disaster in Canadian history thus far.

## Introduction

**Figure 1:**
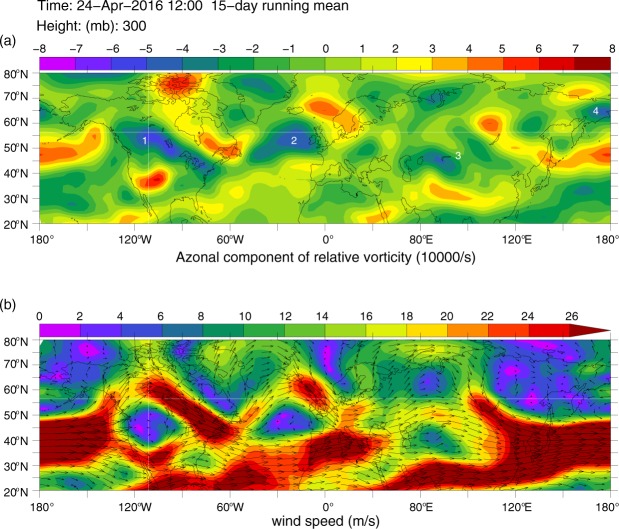
Geographic distribution of important NH atmospheric parameters associated with wave-4 meridional velocity structure (see text). (**a**) the map of relative vorticity, *RV*_300_, in 10^5^ *s*^−1^$.$ The white digits mark the wave-4 related system of anticyclonic circulations, which formed over (1) western Canada, (2) eastern North Atlantic/western Greenland/British Islands, (3) western Siberia/Mongolia, and (4) Russian Far East. (**b**) the map of the vector field of the extratropical atmospheric jets in *ms*^−1^. Both atmospheric parameters are shown at 300 hPa during the 15-day running mean interval from 17^th^ April to 1^st^ of May on the eve of the Alberta 2016 wildfire, based on daily NCEP-NCAR reanalysis data^13^. In both panels, the intersection of the horizontal and vertical straight lines marks the location of the city of Fort McMurray. Created with Ferret v7 (http://ferret.pmel.noaa.gov/Ferret/).

The Alberta Wildfire (hereafter, the Fire) ignited on May 1, 2016 southwest of the city of Fort McMurray. By the end of May, the Fire had left about 590,000 hectares of burned areas, destroyed 2,400 buildings, and led to the largest wildfire evacuation in Albertan history (~90,000 residents affected). The provincial officials declared the Fire to be “under control” only on July 5. This makes the Fire the costliest disaster in Canadian history, total damage reaching as high as Can$4.7 billion^1^. Here we show that the Fire was preceded in late April and accompanied then in May-June by a persistent quasi-stationary high-amplitude planetary wave in the field of the mid/high troposphere meridional velocity with zonal wave number *m* = 4 (hereafter, wave-4) that dominated in the middle and sub-polar latitudes of the northern hemisphere (NH). This wave embraced the whole latitude circle and featured a chain of four closely linked anticyclonic circulations, which were characterized by very high negative values of relative vorticity (hereafter, RV) inherent in very strong NH anticyclones. The above-mentioned anticyclonic circulations formed over western Canada, eastern North Atlantic/western Greenland/British Islands, Siberia/Mongolia, and the Russian Far East (hereafter, the wave-4 related anticyclonic system, wave-4AS). These anticyclonic circulations are marked, respectively, by labels 1 to 4 in Fig. 1a that maps RV at 300 hPa, *RV*_300_, averaged over the 15-day running mean interval from 17^th^ April to 1^st^ of May on the eve of the Fire. Figure 1b reveals high waviness of the extra-tropical jets for the same 15-day running mean interval, apparently caused by and topologically linked to the discussed above wave-4AS.

The Fire happened during a very strong El Niño 2015–2016^2,3^. Such events are considered among the main drivers of the NH wildfires as they usually bring anomalously warm and tinder-dry conditions in corresponding regions^4–11^. In this connection, the El Niño event 2015–2016 is widely seen as one of the main causal factors for the Fire^12^. Here we show that spatial structure and high intensity of some large-scale atmospheric circulations occurring directly in the mid- and sub-polar latitudes of the NH could be another one important factor regulating mid-latitude and sub-polar wildfire activity. The high-amplitude (resonant, as it will be shown in Results) wave-4 structure that reined in the mid/high troposphere of the NH extra-tropics in April-May 2016 can serve as an example of such circulations. Importantly, April 2016 is not the only case of the resonant amplification of wave-4. Also, Aprils 1980, 1983, 2012, and August and September 2017 are discussed in Results, and Discussion. In recent decades, we note also a considerably increasing frequency of occurrences of resonant amplification of this and several other mid- and sub-polar latitude planetary waves with zonal wave wave numbers 4 to 8 and associated weather extremes, which could favor the wildfire weather conditions in the NH extra-tropics. Tracking of such strongly amplifying large-scale planetary waves on 10-day-to-monthly time scale should be gradually introduced into the practice of the wildfire managers and forecasters, because the low-frequency nature of these waves favors their detection at relatively long lead-times (i.e. beyond 10 days) which could potentially improve wildfire forecasts, and because the spatial structure of these resonant waves can dictate locations of very strong anticyclonic circulations associated with them, which support noticeable downward vertical motions, inhibiting middle and high cloudiness, suppressing precipitation and supporting warm and dry conditions at the surface, favoring the wildfire ignition. We discuss this issue at length in Results. Several informative parameters in this context of the large-scale planetary waves are discussed in Results.

## Results

### Surface Conditions (Temperature, Soil Moisture)

**Figure 2:**
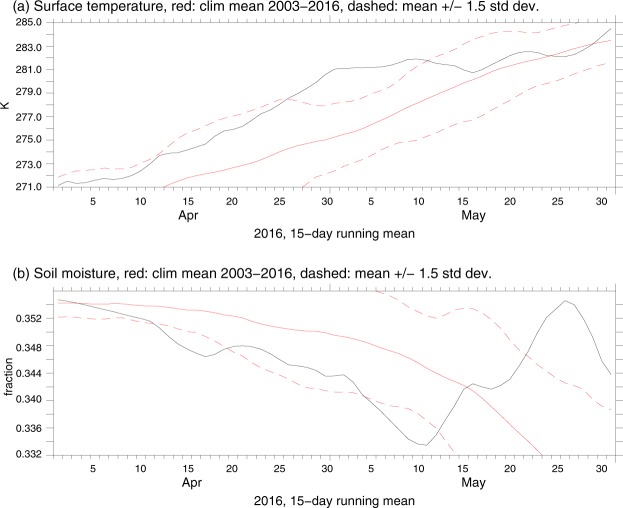
15-day running mean (with central dates of corresponding 15-day intervals marked in the x axis) of the surface temperature in K (panel a) and soil moisture (non-dimensional) in the upper (0–10 cm) soil layer (panel b) over Alberta for April-May 2016 (black curves) in comparison with the 2003–2015 climatology for both variables (solid red curves in both panels), with corresponding 1.5 SD marked by dashed red curves, based on daily NCEP-NCAR reanalysis data^13^.

Figure 2 shows the evolution of the surface temperature *T*_*s*_ (Fig. 2a) and soil moisture *S*_*w*_ (Fig. 2b) over Alberta (110°W–120°W, 50°N–60°N), through April-May 2016. The figure is plotted using time series of the 15-day running means of these variables that we calculated based on daily NCEP-NCAR reanalysis data^13^. As seen in Fig. 2, both variables experienced pronounced trends - positive for *T*_*s*_ and negative for *S*_*w*_ - in late April/early May over Alberta, having been strongly anomalous for that period (beyond 1.5 SD relative to 2003–2015 climatology), and pointing out at unusually warm and tinder-dry conditions in Alberta. A low reliability of the reanalysis data on *S*_*w*_ before the year 2000^14^ did not allow us to track this variable over the total 1980–2016 time range, and we performed a comparison of the 2016 data on *S*_*w*_ over Alberta with the 2003–2015 climatology. The characteristics of the NCEP-NCAR 2003–2015 *S*_*w*_ climatology in the upper soil layer for April-May over Alberta in Fig. 2 compare well with the respective ERA Interim *S*_*w*_ climatology^15^ in the surface layer, while for April-May 2016 ERA Interim data on *S*_*w*_ are noticeably lower than those of the NCEP-NCAR shown in Fig. 2 (cf. Figure 2 with Fig. S1 from SI), suggesting highly abnormal, much drier, conditions at the surface over Alberta through April-May 2016 according to the ERA Interim reanalysis. The ERA Interim data on *T*_*s*_ over Alberta for 2003–2015 April-May climatology and for April-May 2016 agree well with corresponding NCEP-NCAR data.

### Atmospheric Conditions (Geopotential Height, Potential Vorticity, Meridional Wind and Wave Amplitudes)

**Figure 3:**
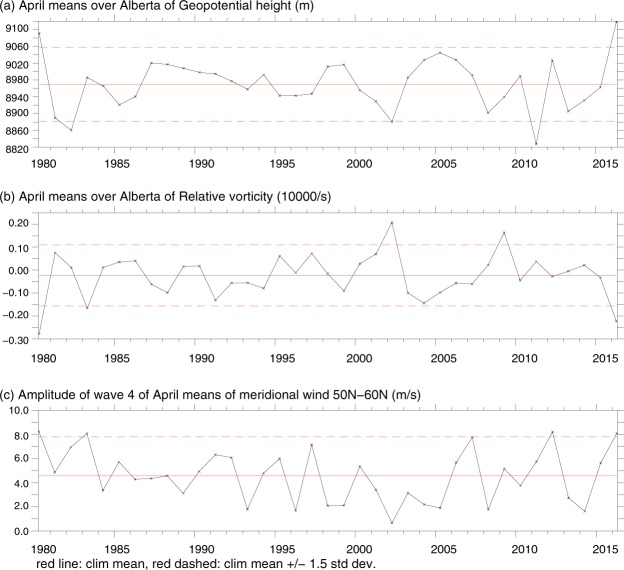
1980–2016 time series of monthly geopotential height *H*_300_, relative vorticity *RV*_300_ and the amplitude of wave-4 Fourier component of the meridional velocity at 300 hPa (black curves) for April averaged over the Alberta region (110°W–120°W, 50°N–60°N), based on daily NCEP-NCAR reanalysis data^13^. The horizontal solid and dashed red lines in all panels mark the 1980–2015 climatological mean and corresponding ±1.5 SD from the mean.

The year 2016 was one of the two strongly anomalous years (2016 and 1980) within the 1980–2016 time range over Alberta in April, with high positive and negative anomaly at the equivalent barotropic level (EBL) 300 hPa, respectively, for geopotential height *H*_300_ (Fig. 3a) and *RV*_300_ (Fig. 3b). This indicates strong anticyclonic circulation there, as compared to all other years of 1980–2016, favoring dry and warm conditions at the surface and increasing the danger of wildfires over Alberta in April of those two years. 2016 also enters a set of four years (1980, 1983, 2012 and 2016) that feature in April, according to monthly NCEP-NCAR reanalysis data^13^, the highest amplitudes of monthly wave-4 Fourier component of the meridional velocity at 300 hPa among all Aprils 1980–2016, exceeding 1.5 SD of the 1980–2015 climatology (Fig. 3c).

**Figure 4:**
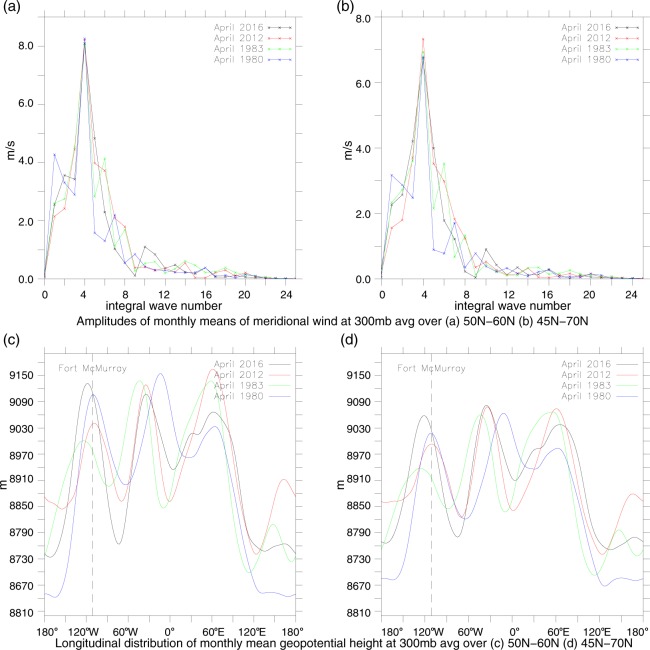
The amplitudes of the Fourier components, up to *n* = 24, of monthly meridional velocity at 300 hPa and the longitudinal distributions of monthly *H*_*300*_ for Aprils of the years of 1980, 1983, 2012 and 2016, based on respective NCEP-NCAR reanalysis data^13^. Panels (a) and (b) demonstrate a dominance of wave-4 Fourier component over all other Fourier components of the meridional velocity averaged, respectively, over 50N–60N and 45N–70N, while panels (c) and (d) show the longitudinal distribution of *H*_*300*_ averaged over the same latitudinal ranges 50N–60N (**c**) and 45N–70N (**d**), with one of the maxima reached over western Canada.

**Figure 5:**
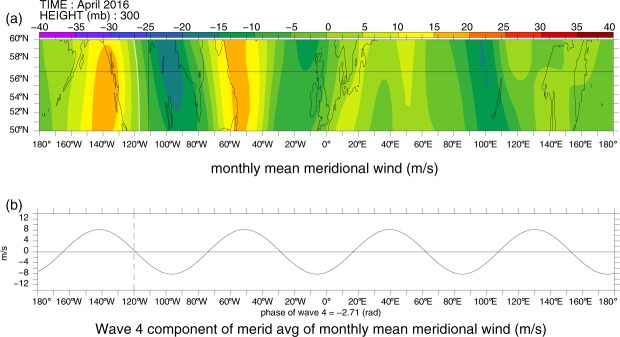
(**a**) The longitudinal distributions of monthly meridional velocity at 300 hPa, *V*_300_, within the 50N–60N belt, and (**b**) its wave-4 Fourier component averaged over that belt, for April 2016. In (**a**), the black cross marks Fort McMurray, the white line marks *V*_*300*_ = 0 at ca. 120° W. Created with Ferret v7 (http://ferret.pmel.noaa.gov/Ferret/).

Moreover, in those four years monthly wave-4 for April was the dominant one in the mid- and sub-polar latitudes of the NH. This conclusion is supported by Fig. 4 that pictures the amplitudes of the Fourier components, up to *n* = 24, of monthly meridional velocity at 300 hPa ((a) and (b)) and longitudinal distribution of monthly *H*_300_ ((c) and (d)), averaged over the 50N–60N ((a) and (c)) and 45N–70N ((b) and (d)). Figure 4a,c indicates that in all four years wave-4 Fourier component had the highest amplitude among the total planetary wave field, both in mid- and sub-polar latitudes of the NH, with four high ridges of *H*_300_ (see Fig. 4c,d) accompanying four strong anticyclonic circulations with highly negative ${{RV}}_{300}$ in the wave-4AS shown in Fig. 1a. Furthermore, Fig. 4c,d attest that in all four Aprils western Canada was located just within one of these anticyclonic circulations. This was caused by the occurrence of an active thermal source of negative RV in the considered region, due to a sharp longitudinal gradient of the surface temperature between oceanic and land masses across a narrow strip of the eastern Pacific coast. In combination with a strong orographic source of RV of the same sign caused by steep Rocky Mountains massive located in the region, the above-mentioned active thermal source of RV appeared to be the most powerful one within the considered two latitudinal belts at that time, dictating in this way a position of the governing zero phase at ca. 120°W for the wave-4 longitudinal distribution along both latitudinal circles, fully in line with Haurwitz theory^16^ of atmospheric sources of RV. It is instructive to plot a total monthly mean meridional velocity at 300 hPa, *V*_300_, together with its wave-4 component for April 2016 in the 50N–60N latitudinal belt (see Fig. 5).

Panel (a) of Fig. 5 shows the geographic map of *V*_300_ within the belt, while the panel (b) of this figure depicts the longitudinal distribution of its wave-4 Fourier component averaged over the belt. Note that the location of the zero phase of the *V*_300_ at ca. 120°W (see white curve in panel (a)) in its longitudinal distribution matches rather well the position of zero phase in the longitudinal distribution of its wave-4 Fourier component averaged over the belt (see dashed line in panel (b)). This is due to the dominant contribution of wave-4 to the total spectrum of *V*_300_ shown in Fig. 4a.

On the other hand, a comparison of Figs 4c and 5a,b attests that the positions of the zero phase in the wave *V*_300_ and wave-4 longitudinal distributions over western Canada at ca. 120°W correspond well to the location of the crest (maximum) in the longitude distribution of the geopotential height *H*_300_. This can be explained by a strong dynamical coupling of *V*_300_ and *H*_300_ fields, on the strength of hydrostatic balance and quasi-geostrophic relations (see equation (1)) below). This results, in turn, in the anomalously high pressure over Alberta throughout the total depth of the mid and lower troposphere in April 2016^13^, due to a very strong anticyclonic branch of RV (Fig. 3a,b), which entailed a strong downward vertical motion in the mid- and lower troposphere. These could favor a pronounced adiabatic warming of the mid- and lower troposphere air, accompanied by a decrease in high/mid cloud amount and drying/warming of the surface^17^. This led to a rather high wildfire hazard situation over Alberta in April 2016. Let us note that we discuss here persistent (with 10-day to monthly time scales), resonant-type planetary waves, which anticyclonic wings operate in the situations of nearly absence of cumulonimbus clouds, with lightning strikes as rare events.

### Conditions that lead to amplified planetary wave-4 (resonance)

The anomalously high amplitudes of wave-4 and wave *V*_300_ shown in Fig. 5 are due to the mechanism of quasi-resonant amplification (QRA) of quasi-stationary planetary waves first proposed in^18^ and developed further in^19–21^. This mechanism is considered here on a particular example of quasi-stationary planetary wave-4. Given the quasi-geostrophic coupling between *V*_300_ and *H*_300_, QRA led also to a high amplitude of the respective *H*_300_ wave. The essence of the QRA mechanism is trapping (locking) and corresponding strong (resonant) amplification of the energy of the mid/high troposphere quasi-stationary planetary waves with non-dimensional zonal wave numbers *m* = 4–8 within specific mid-latitude and sub-polar waveguides. This happens due to drastically diminished exchange of the trapped wave action (energy) between mid- and tropical, and sub-polar and polar latitudes caused by a strong reflection of these waves at the lateral (meridional) boundaries of the waveguides^18^. The occurrence of the above-mentioned waveguides is strictly regulated by a set of certain necessary conditions, which the latitudinal distribution of the zonally averaged zonal winds should meet^18–21^. We have previously implemented and documented an automated QRA-detection-scheme developed in^20,21^ which identifies the presence of the QRA resonance in the mid- and/or sub-polar latitude ranges. As we noted earlier, the frequency of the QRA events and associated weather extremes noticeably increased over recent decades, possibly due to anthropogenic forcing^22^.

**Figure 6:**
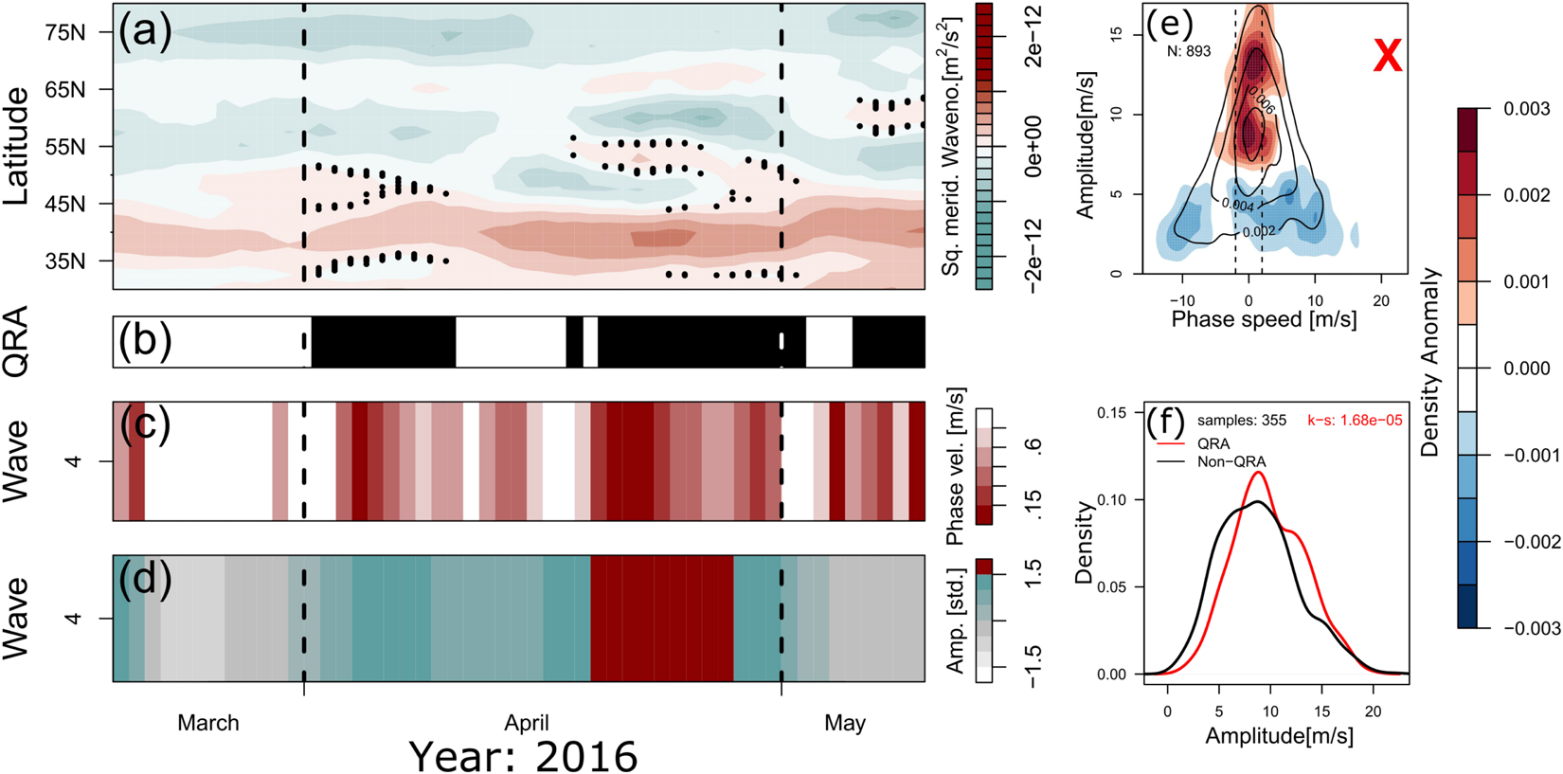
Application of the automated QRA-detection-scheme^20,21^ for wave-4 at 300 hPa. Panels (a–d) correspond to March-April-May (MAM) 2016 and panels (e,f)–to MAM of years 1979–2016. (**a**) the detected waveguides (marked by black dots) for quasi-stationary, QRA wave-4. (b) the detected duration of the QRA periods for QRA wave-4 (marked in black). (**c**) the QRA wave 4 phase speed (in m/s) marked in colors, with corresponding color legend in the y-axis. (**d**) the amplitude of QRA wave-4 in units of standard deviation marked in colors, with respective color legend in the y-axis. (**e**) changes in the probability density function of amplitude vs. phase speed for different wave-4 components during QRA periods as compared to the non-resonance days (red and blue filled color contours designate, respectively, positive and negative changes in amplitude, with appropriate color legend shown in the y-axis). Solid black curves in panel (e) depict corresponding 1979–2016 MAM climatology. (**f**) the probability density function distribution for the wave-4 amplitudes during QRA periods (red curve) vs. non-resonance days (black curve), over 1979–2016 time span. When QRA conditions are granted the quasi-stationary component of wave-4 is significantly amplified. For more details, see^18,20,21^.

When we applied the QRA-detection-scheme to the NH extra-tropics for March-April-May (MAM) 2016, we found that QRA resonance conditions for wave-4 were present in MAM 2016 in the 35°N–70°N belt and the scheme predicted its high amplitudes within this time range (see Fig. 6). We analyzed 15-day running means of the meridional velocity in the mid- and sub-polar latitudes of the NH at 300 hPa for the 15-day time periods with central dates from 24 March 2016 to 7 May 2016 (see Fig. 6). Here, slowly moving, quasi-stationary components of wave-4 had significantly higher amplitudes during QRA events, while fast-moving components demonstrated the opposite tendency. In accordance with this finding, the probability density function distribution for the wave-4 amplitudes is narrower and steeper, and has higher maximum during QRA days, as compared to non-resonance periods. According to the results of our calculations, the QRA quasi-stationary wave-4 developed within the corresponding waveguides (cf.^18,20,21^) in early April at latitudes 35°N–55°N, and then again in the second half of April around latitudes 40°N, 55°N and 65°N (see Fig. 6a). Both detected events are associated with low phase speed and very high amplitude (higher than 1.5 SD from 1980–2016 climatology) of this wave.

In agreement with what we discussed in the beginning of this section of Results the meridional exchange of atmospheric planetary wave action (energy) between tropics and the extra-tropical latitudes was small during the periods of occurrence (marked in black in Fig. 6b) of high-amplitude quasi-stationary QRA wave-4 in MAM 2016. Fig. S2 in SI shows the example of such situation that emerged on the eve of the Fire. In this figure, locking of the resonant QRA wave-4 is traced by close to zero values of the meridional velocity at 300 hPa over subtropical belt at ca. 25°N-35°N. Under these circumstances, the above-mentioned wave-4AS system of four high-amplitude anticyclones began to drive - in concert with a very strong El Niño 2015–16 – highly fire-hazardous, tinder-dry and warm temperature conditions over respective regions of the NH marked by labels in Fig. 1a so unusually early, already in April (see Fig. 2 and Fig. S1). This just favored the ignition of strong wildfires at the onset of May not only over Canada, but also over a broad 45N–70N belt of the NH. Indeed, May-June of 2016 featured outbreaks of simultaneous, closely linked (concatenated) forest and steppe wildfires favored by wave-4AS (see, e.g.,^23,24^).

### Statistical correlations between Canadian wildfire activity and large-scale atmospheric weather/climate characteristics

To get an estimation of the depth of correlation between the Canadian wildfire activity in April with ENSO we show a table (hereafter, Table 1) of Pearson coefficients of correlation, *r*, of 3-monthly running means (ONI-NDJ to ONI-MAM) of the ONI ENSO Index^2,3^ with monthly numbers of the wildfires in April (MNWA) over Alberta (FiresAB) and all-Canada (FiresCA) from^25^, based on 1980–2016 statistics of ONI and MNWA.

**Table 1:** Pearson coefficients of correlation between the Oceanic Niño Index (ONI^2,3^), and monthly number of wildfires in April (MNWA) over Alberta (FiresAB line) and all-Canada (FiresCA line) according to^25^, both ONI and MNWA being calculated based on 1980–2016 statistics. In each column, the results are shown of corresponding calculations performed with the use of 3-monthly moving average of ONI, from November-December-January (ONI-NDJ) of the previous year up to April-May-June (ONI-AMJ) of the current year.

ENSOIndex	ONI-NDJ	ONI-DJF	ONI-JFM	ONI-FMA	ONI-MAM	ONI-AMJ
FiresAB	0.307368	0.324309	0.323246	0.29865	0.249624	−0.0219518
FiresCA	0.228647	0.251896	0.267226	0.248496	0.187463	−0.00950143

Each column in Table 1 shows the values of *r* that we obtained with the use of respective 3-monthly moving average value of ONI, from November-December-January (ONI-NDJ column) of the previous year up to March-April-May (ONI-MAM column) of the current year.

As seen from Table 1 the maximum coefficient of correlation, *r*_*max*_, between ONI and MNWA is reached, when using in respective calculations ONI-DJF for the Alberta Province (*r*_*max*_ ≈ 0.324), and ONI-JFM for Canada as a whole (*r*_*max*_ ≈ 0.267). This indicates a rather low correlation between ONI and MNWA. The conclusion is supported by the analysis of the individual years. E.g., according to^25^ the numbers of strong wildfires over Canada in Aprils of 1980, 1987, 1991, 2006 and 2010 with weak and moderate El Niño^2,3^, as well as in April of 1988 with strong La Niña^2,3^, exceeded those in Aprils of 1982–1983 and 1997–1998 with very strong El Niño. This points to an important contribution to MNWA from specific hydrological and vegetation cover conditions over a given region in a particular year/month (see, e.g.,^26^), as well as the amount of snowfall in the previous winter and snow melting in the spring’s eve. Here we show that one more important factor regulating MNWA can be the large-scale structure of the atmospheric circulations over the mid- and sub-polar latitudes of the NH.

In this context, our calculations reveal a significant correlation between MNWA and monthly *H*_300_ averaged over Alberta and all-Canada for Aprils of 1980–2016, with *r* about 0.67 for all-Canada and 0.62 for Alberta. On the other hand, our calculations show that due to the quasi-geostrophic relation^27^

1 $${V}_{300}(x,\,y)=\frac{g}{f}\frac{\partial {H}_{300}(x,\,y)}{\partial x}$$

and the hypsometric equation^27^

2 $${\bar{H}}_{300}(y)\approx \frac{R}{g}{\tilde{T}}_{0}^{{H}_{300}}(y)\mathrm{ln}\,\frac{{\bar{p}}_{0}(y)}{300},$$

there exists a high correlation (r ~ 0.9–0.95) of the values of *H*_300_(*x*, *y*) with a certain linear combination, *LC*_*N*_(*x*, *y*), of ${[\bar{T}]}_{0}^{{H}_{300}}$(y) and a set of the first *N* large-scale components, *F*_*n*,*V*_(*x*, *y*), of *dV*_300_/dx Fourier decomposition. Here, *g* is the acceleration due to gravity, *f* is the Coriolis parameter, *R* is the gas constant for the air, while ${\bar{p}}_{0}(y)$ is a zonally averaged surface pressure in hPa^13^, ${[\bar{T}]}_{0}^{{H}_{300}}(y)$ is a zonally averaged temperature pressure-weighted over the (${\bar{p}}_{0}(y),\,300$) range^13^, and ${\bar{H}}_{300}(y)$ is the zonal average of ${H}_{300}(x,y)$. With the use of equations (1) and (2) the above-mentioned linear combination *LC*_*N*_(*x*, *y*) is described by:

3 $$L{C}_{N}(x,\,y)=\sum _{n=1}^{N}-\frac{f}{g}\frac{{a}^{2}}{{n}^{2}}{F}_{n,V}(x,\,y)+\frac{R}{g}{[\bar{T}]}_{0}^{{H}_{300}}(y)\mathrm{ln}\,\frac{{\bar{p}}_{0}(y)}{300},$$

where *a* is the Earth’s radius. In our calculations, we put *N* = 9, as the number of the main components (cf. Fig. 4a,b) in the meridional velocity longitudinal Fourier decomposition. A significant Pearson correlation (with *r* about 0.6–0.7) is found between MNWA and 〈*LC*_*N*_(*x*, *y*)〉_*A*_ (with 〈*X*〉_*A*_ as the area average of *X* over the region). Importantly, Pearson correlation coefficients appear to be low (below 0.2) between MNWA and the amplitude of any individual wave from the above planetary wave set. This emphasizes the importance of taking into account, in the general case, not only the amplitudes but also the phases of the considered waves and the need for the full wave-ensemble description of the main waves when calculating the above correlation. Note, that the weighing factor *n*^2^ in the first summand in the r.h.s. of equation (3) accounts for the application in our calculations of the geostrophic approximation (1). Omitting 1/*n*^2^ weighing factor in equation (3) reduces Pearson correlation coefficients between 〈*LC*_*N*_(*x*, *y*)〉_*A*_ and MNWA to 0.4 as the maximum.

### Comparison of the years of 1980, 1983, 2012 and 2016 in the context of the large-scale NH atmospheric parameters and the link of these parameters to the Canadian wildfire activity in Aprils of these years

Above, we mentioned 4 years, 1980, 1983, 2012 and 2016, with strongly anomalous heights *H*_300_ of 300 hPa surface over western Canada (in 1980 and 2016) and high amplitudes of planetary wave-4 in the mid- and sub-polar latitudes of the NH (in all 4 years) in Aprils (see Fig. 3). In the above text, we discussed basically April-May 2016. Keeping in mind very high amplitudes of wave-4 demonstrated by 1980, 1983 and 2012 in Aprils, which are quite comparable with that in April 2016, we may assume that the mechanism of wave-4 generation could be the same (QRA) in all 4 years. To check this hypothesis we plotted three additional figures (see Figs S3, S4 and S5 in the SI), analogous to panels (a)–(d) in Fig. 6. As seen from these figures, wave-4, in fact, experienced a strong quasi-resonant amplification, specifically in the beginning and in the end of Aprils 1980, 1983 and 2012 as in April 2016 (cf. Fig. 6). Secondly, as far as the height *H*_300_ of the 300 hPa surface over Canada in Aprils appeared to be rather closely correlated with the number of the Canadian wildfires (see above), we may assume that the abnormally high altitude of the 300 hPa surface in April 1980 over Alberta could be accompanied by a markedly high number of wildfires in the Province in that month. In this connection, according to^25^ data, 1980 adds to a set of three years (2006, 2010 and 1980) with the highest monthly numbers of wildfires over Alberta in Aprils. These numbers (350 in 2006, 342 in 2010 and 302 in 1980) were all above mean + 1.5 SD = 261 for the 1980–2016 climatology. April 2016 is the fourth one among the Aprils from the 1980–2016 range of years, with 284 monthly numbers of fires, also markedly above mean + 1.5SD 1980–2016 climatology^25^.

Further, on the strength of a very high correlation between *H*_300_(*x*, *y*) and *LC*_*N*_(*x*, *y*) (with r ~ 0.9–0.95), we might reformulate a close relationship between the area-averaged *H*_300_ over Alberta and MNWA in Aprils in terms of 〈*LC*_*N*_(*x*, *y*)〉_*A*_ and MNWA, and include 〈*LC*_*N*_(*x*, *y*)〉_*A*_ into a set of possible tracers for MNWA. Then, because of low values of both *H*_300_(*x*, *y*) and *LC*_*N*_(*x*, *y*) in Aprils 1983 and 2012 (noticeably below mean + 1.5 SD from the 1980–2016 climatology, see Fig. 3a), we can anticipate a rather low values of MNWA in Aprils of these two years. Indeed, MNWA was only 86^25^ over Alberta in April 1983, and in April 2012 it was 122^25^, which is lower than 1980–2016 climatology (123, according to^25^). This happened, despite very high values of the wave-4 amplitude in those two Aprils (see Fig. 3c). This fact indicates, again, that a full set of main planetary waves with phase positions and regional thermodynamical conditions should be accounted for when estimating correlations between MNWA and climate characteristics.

Statistical estimations of the numbers of wildfires do not always track the area burnt and strongly depends on the prescribed minimum size. In this connection, with ca. 590,000 hectares burnt the Fire is Canada’s fourth largest fire on record^25^ contributing to the ca. 634,000 ha burnt in Alberta in 2016^25^. April is usually the start of the fire season, where in the anomaly years 770 ha (2010; 5392 ha in May) or 3575 ha (2006; 3193 ha in May) forests were burnt in Alberta, whereas on average ca 800 ha (74000 ha) were burnt in April (May) in 1990–2015^25^. Burnt area is not only influenced by fire weather conditions, including wind, but also orography, fuel load, flammability of the dead and living biomass, and forest structure. Therefore, MWNA do not necessarily lead to maximum area burnt, with the exception of the Fire which contributed approximately 96% (42%) to the annual area burnt in Alberta (Canada) in 2016.

## Discussion

In our analysis, we find that the anomalously high-amplitude quasi-stationary QRA wave-4 in April-May 2016 could be one of the factors favoring the ignition and geographic localization of the Fire in May-June 2016. This wave-4 could also trigger the occurrence of wave-4AS in April-June 2016, contributing to the generation of strong forest and steppe wildfires over the land masses in respective regions^23,24^ and favoring the wavy structure of the extratropical jets in the NH.

The approach proposed here allows us to estimate a probability of the climate-forced trends of regional MNWA linked to the change of “global” parameters, ${[\bar{T}]}_{0}^{{H}_{300}}$ and 〈*LC*_*N*_(*x*, *y*)〉_*A*_. Analogous estimations can be applied to other months and land areas susceptible to high wildfire activity. This might be especially useful in the studies on probability of strong wildfires and their trends under different future climate change scenarios.

Let us note that Lagerquist *et al*. (2017) developed recently a first machine-learning model for the goal of extreme fire weather prediction over Northern Alberta, using regional self-organizing maps (SOMs), with sea-level pressure and 500 hPa height as the predictors^28^. Employing the analogous weather predictors as in^28^, we use the information on the large-scale structure of dynamic and thermodynamic atmospheric fields in the NH middle and sub-polar latitudes.

We may speculate that recently observed slow-down of the NH mid-latitude zonal circulation due to Arctic amplification^29^ could increase the occurrences of the enlarged 〈*LC*_*N*_(*x*, *y*)〉_*A*_ leading to the growing danger of extreme wildfires. This hypothesis is supported by the fire weather situation that was observed over the NH mid- and subpolar latitudes in August-September 2017. A very high-amplitude quasi-stationary (QRA) wave-4 reined there again in the middle and high troposphere^13^, being accompanied by the wave-4AS (see Fig. S6), with the fire weather situation featured in that case by the exceptional in number extreme wildfires in western Greenland^30^.

In the end let us note that tracking of strongly amplified QRA planetary waves on 10-day-to-monthly time scale using the QRA-detection-scheme developed in^20^,^21^, and applying equations (1)–(3) for the estimation of the correlations between 〈*LC*_*N*_(*x*, *y*)〉_*A*_ and MNWA suggested here, could be rather useful for and gradually be incorporated into the practical work of the wildfire forecasters. This is because the spatial structure of these resonant waves can dictate locations of very strong anticyclonic circulations, favoring high wildfire hazard in corresponding regions.

## Methods

This section provides an overview of the basic methods used here to obtain the main results. The geographic maps of the azonal component of the relative vorticity and the vector field of the extratropical atmospheric jets at 300 hPa pressure level shown in Fig. 1a and b, respectively, as well as the graphs for the surface temperature and soil moisture over Alberta (Fig. 2a and b) are created applying the appropriate time series of the 15-day running means of these variables calculated based on daily NCEP-NCAR reanalysis data for April-May 2016^13^. Figures 3c, 4a,b and 5a,b are plotted using a Fourier decomposition method to the respective longitudinal distributions of monthly meridional velocity at 300 hPa level in the NH based on daily NCEP-NCAR reanalysis data^13^ for Aprils of different years over the 1980–2016 time range. Figure 6a–f demonstrate the results obtained here, applying the physical mechanism and the methodology of the Quasi-Resonant-Amplification (QRA) of the quasi-stationary atmospheric planetary waves proposed in^18^, to planetary wave-4 at 300 hPa for March-April-May of the years covering 1979–2016 with the usage of the automated QRA-detection scheme developed in^20,21^. In Results and  Discussion we describe the results of our analysis, in terms of the Pearson correlation coefficients, of the statistical relationships between wildfire activity in the fire-susceptible NH regions and the parameters of the NH large-scale planetary-wave atmospheric circulation and the ENSO indices.

## Conclusions

We showed noticeable contribution of the large-scale planetary wave circulations in the mid/high troposphere of the NH extra-tropics to the ignition of strong forest and steppe wildfires over Canada. We found that the anomalously high-amplitude QRA wave-4 was one of the important factors favoring the catastrophic wildfire in the Alberta Province of Canada in May-June 2016. This wave-4 also triggered the occurrence in April-June 2016 of the chain of strong anticyclonic circulations over western Canada, eastern North Atlantic/western Greenland/British Islands, Siberia/Mongolia, and the Russian Far East. These, in turn, led to the ignition in respective land areas of synchronous strong wildfires encircling the NH and favored the observed wavy structure of the NH extratropical jets. Generally, the results of our statistical analysis indicate that strong wildfires over Alberta and all-Canada appear to be noticeably linked to a set of large-scale quasi-stationary resonant planetary waves in the NH extra-tropics.

## Electronic supplementary material

Supplementary Information

## Data Availability

All data used for this research was downloaded from public sources, as referenced throughout the text. Data and processing scripts are stored in PIK’s long-term archive, and will be made available to interested parties upon request. All plots and maps shown were created by the authors.
