# Supplementary material for: Alberta wildfire 2016: Apt contribution from anomalous planetary wave dynamics
Source: Sci Rep. 2018 Aug 17;8:12375. doi: 10.1038/s41598-018-30812-z (PMC6098075; doi:10.1038/s41598-018-30812-z)
Supplement: Supplementary file 1 — Supplementary Information [file 41598_2018_30812_MOESM1_ESM.doc]

Alberta wildfire 2016: Apt contribution from anomalous planetary wave dynamics.

Vladimir Petoukhov, Stefan Petri, Kai Kornhuber,

Kirsten Thonicke, Dim Coumou, and Hans Joachim Schellnhuber

Potsdam Institute for Climate Impact Research (PIK), Member of the Leibniz Association, P.O. Box 60 12 03, D-14412 Potsdam, Germany, Vrije Universiteit, Amsterdam, Netherlands, Stockholm Resilience Centre, Stockholm University, 10691 Stockholm, Sweden

To whom correspondence may be addressed. E-mail: john@pik-potsdam.de or petukhov@pik- potsdam.de

**Supplementary Information (SI).**

**
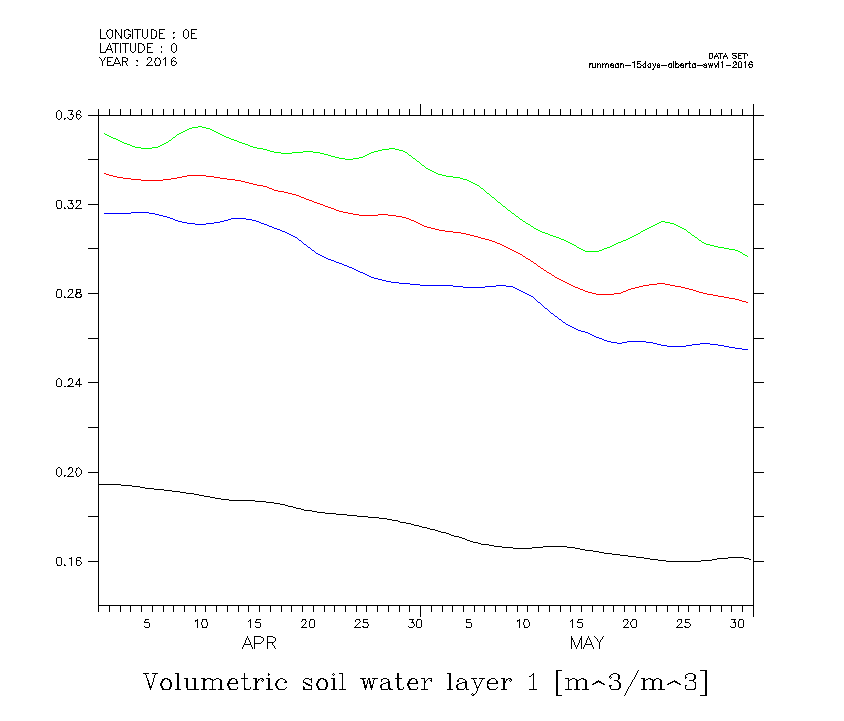
**

**Fig. S1 April-May soil moisture in the surface layer (0-7 cm) over Alberta according to the ERA Interim reanalysis data (15). The black curve corresponds to the year of 2016, while the red curve relates to the 2003-2015 climatology, with respective 1.5 SD shown by the green and blue curves. All the data shown are the 15-day running means through April-May.**


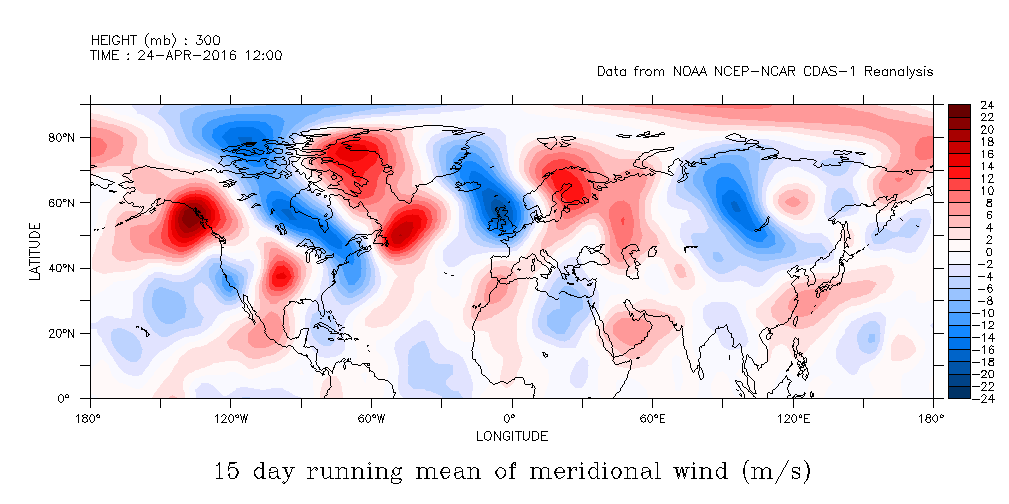


**Fig. S2. NH geographic distribution of the 15-day mean of the quasi-stationary meridional velocity at 300 hPa, with 24 April 2016 as the central date on the eve of the start of the Alberta Wildfire 2016 ignition on May 1, based on NCEP-NCAR daily reanalysis data (13). A high-amplitude, QRA wave 4 pattern is clearly traced over the 35N-70N mid- and subpolar-latitude belt, with only a minor influence on the pattern from the tropics, due to low values of the meridional wind velocity over subtropics, approx. in 25N-30N latitudinal range. Created with Ferret v7 (**[**http://ferret.pmel.noaa.gov/Ferret/**](http://ferret.pmel.noaa.gov/Ferret/)**).**

**Figure S3: Analogous to the panels (a)-(d) in Figure 6 of the main text, but for the year of 1980**.

**Figure S4: Analogous to the panels (a)-(d) in Figure 6 of the main text, but for the year of 1983.**

.

**Figure S5: Analogous to the panels (a)-(d) in Figure 6 of the main text, but for the year of 2012**


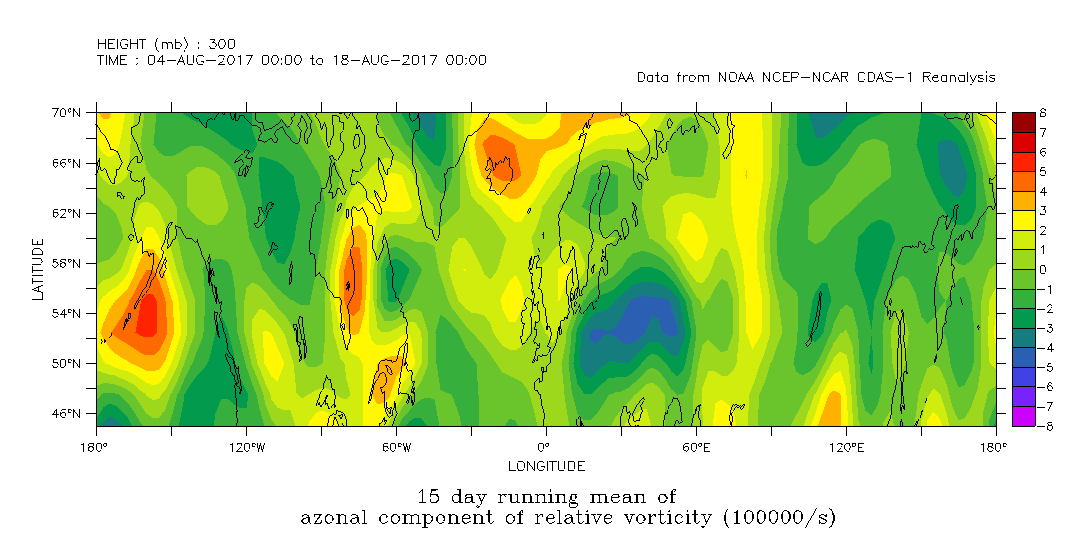


**Fig. S6. The relative vorticity at 300 hPa in the mid- and sub-polar latitudes of the NH over the 15-day mean interval from 4^th^ to 18^th^ August 2017 as the example of the atmospheric circulation in August-September 2017 featured by the exceptional in number extreme wildfires in Greenland, based on daily NCEP-NCAR reanalysis data (13). Created with Ferret v7 (**[**http://ferret.pmel.noaa.gov/Ferret/**](http://ferret.pmel.noaa.gov/Ferret/)**).**
